# Outage Probability Minimization for Energy Harvesting Cognitive Radio Sensor Networks

**DOI:** 10.3390/s17020224

**Published:** 2017-01-24

**Authors:** Fan Zhang, Tao Jing, Yan Huo, Kaiwei Jiang

**Affiliations:** School of Electronic and Information Engineering, Beijing Jiaotong University, Beijing 100044, China; tjing@bjtu.edu.cn (T.J.); yhuo@bjtu.edu.cn (Y.H.); kwjiang@bjtu.edu.cn (K.J.)

**Keywords:** cognitive radio, energy harvesting, sensor networks, Markov decision process

## Abstract

The incorporation of cognitive radio (CR) capability in wireless sensor networks yields a promising network paradigm known as CR sensor networks (CRSNs), which is able to provide spectrum efficient data communication. However, due to the high energy consumption results from spectrum sensing, as well as subsequent data transmission, the energy supply for the conventional sensor nodes powered by batteries is regarded as a severe bottleneck for sustainable operation. The energy harvesting technique, which gathers energy from the ambient environment, is regarded as a promising solution to perpetually power-up energy-limited devices with a continual source of energy. Therefore, applying the energy harvesting (EH) technique in CRSNs is able to facilitate the self-sustainability of the energy-limited sensors. The primary concern of this study is to design sensing-transmission policies to minimize the long-term outage probability of EH-powered CR sensor nodes. We formulate this problem as an infinite-horizon discounted Markov decision process and propose an *ϵ*-optimal sensing-transmission (ST) policy through using the value iteration algorithm. *ϵ* is the error bound between the ST policy and the optimal policy, which can be pre-defined according to the actual need. Moreover, for a special case that the signal-to-noise (SNR) power ratio is sufficiently high, we present an efficient transmission (ET) policy and prove that the ET policy achieves the same performance with the ST policy. Finally, extensive simulations are conducted to evaluate the performance of the proposed policies and the impaction of various network parameters.

## 1. Introduction

During the last decade, bandwidth demand for the limited spectrum has been greatly increasing due to the explosive growth of wireless services. The current static frequency allocation schemes, with a severe underutilization of the licensed spectrum over vast temporal and geographic expanses [1], cannot support numerous emerging wireless services. This motivates the concept of cognitive radio (CR) [2,3,4], which has been envisioned as an intelligent and promising approach to alleviate the problem of spectrum utilization inefficiency. In CR networks (CRNs), unlicensed secondary users (SUs) opportunistically access the spectrum dedicated to some licensed primary users (PUs) without interfering with the PU operation [5]. Through enabling the CR users to dynamically access the available bands in the licensed spectrum, spectrum efficiency can be improved significantly.

The wireless sensor network (WSN), which is capable of performing event monitoring and data gathering, has been applied to various fields, including environment monitoring, military surveillance, smart homes and other industrial applications [6,7]. Currently, most WSNs work in the license-free band and are expected to suffer from heavy interference caused by other applications sharing the same spectrum. It is therefore imperative to employ CR in WSNs to exploit the dynamic spectrum access techniques, hence giving birth to the CR sensor networks (CRSNs) [8,9]. In CRSNs, in order to guarantee the quality-of-service (QoS) of primary users, it is indispensable for CR sensor nodes to sense the licensed spectrum to ensure that the spectrum is free of primary activities before data transmission. The exclusive operation of spectrum sensing along with subsequent data transmission results in high energy consumption in CRSNs, which traditionally operate powered by batteries. Consequently, one of the looming challenges that threatens the successful deployment of CRSNs is the energy efficiency [6,10].

Energy harvesting (EH) technology, which is used to replenish energy from various energy sources, such as solar, wind and thermal, has been flagged as one of the effective approaches for improving the energy efficiency with more eco-friendliness [11]. Compared with traditional communication devices powered by batteries, EH-enabled devices could scavenge unlimited energy from the ambient environment energy sources, which enable them to operate continuously without battery replacement [6]. This self-sustainable feature is very important because in many situations, periodically replacing or recharging batteries may be inconvenient or even impossible due to various physical restrictions [12]. Besides, powering wireless networks with renewable energy source could also significantly reduce the harmful effects to the environment caused by fossil-based energy. Furthermore, energy harvesting systems can be built inexpensively in small dimensions, which could be a significant advantage in the manufacturing of small communication devices, such as sensor nodes [13]. Recently, apart from traditional energy sources (e.g., solar, wind, thermal), the ambient radio signal is also regarded as a helpful optional source, which can be consistently available regardless of the time and location in urban areas [14]. In light of the above advanced features, applying EH in CRSNs to improve energy efficiency has become increasingly eye-catching recently [10,15,16,17].

In this paper, we consider a time-slotted EH CR sensor network, where the secondary sensor node (also called SU) with a finite-capacity battery has no fixed energy supply and is powered exclusively by energy harvested from the ambient environment. There are multiple tradeoffs involved in the design of the parameters to achieve the optimal system performance of the SU. First, due to the existence of sensing errors, with a longer time allocated for channel sensing, the SU can acquire the status of a licensed spectrum with higher accuracy, such that the performance of the SU may be improved. However, in the slotted operating mode, with more time allocated for channel sensing, less time remains for data transmission, leading to possible performance reduction. Besides, as the amount of transmitting power used upon transmission will affect both the performance and energy consumption, a crucial challenge lies in adaptively tuning the transmission power levels according to the energy replenishment process, as well as channel variation. An overly conservative power allocation may limit the performance by failing to take full advantage of harvested energy, while an overly aggressive allocation of power may cause the energy in the battery to run out and affect the performance of the future time slots. Additionally, different from traditional CR systems with a fixed power supply, the energy consumption on the channel sensing is non-negligible in EH CRSNs; therefore, the problem of designing parameters, which should jointly consider the energy consumption of channel sensing and data transmission, as well as the dynamic battery replenishment process, becomes even more complicated than the traditional CR systems.

The objective of this paper is to minimize the long-term outage probability of the secondary sensor node by adapting the sensing time and the transmit power to the system states, including the battery energy, channel fading and the arrival energy by harvesting. The main contributions of this work are summarized as follows:

1. Considering the status of primary channels, the diversity of channel conditions, the energy replenishment process, as well as the imperfection of spectrum sensing, we investigate the joint optimization of channel sensing and adaptive transmit power allocation to minimize the SU’s long-term outage probability. The above design problem is formulated as a discounted Markov decision process (MDP).
2. We theoretically prove the existence of an optimal stationary deterministic policy and obtain the *ϵ*-optimal sensing-transmission (ST) policy, which specifies the allocation of sensing time and transmission power through using the value iteration in the MDP. Moreover, an interesting structural property regarding the optimal transmission policy is obtained. It is proven that the optimal long-term outage probability is non-increasing with the amount of the available energy in the battery.
3. For a special case where the signal-to-noise (SNR) power ratio is sufficiently high, we propose an efficient transmission (ET) policy with reduced computational complexity. It is theoretically proven that the efficient transmission policy achieves the same performance as the proposed sensing-transmission policy when the SNR is sufficiently high, which has also been validated through computer simulations.
4. We provide extensive simulation results to compare the performance of the sensing-transmission policy and the efficient transmission policy with that of a benchmark policy. It is shown that the proposed sensing-transmission policy achieves significant gains with respect to the benchmark policy, and both the sensing-transmission and the efficient transmission policies converge to the same value in high SNR regions. In addition, the impacts of various system parameters on the performance of proposed policies are also investigated.

The rest of the paper is organized as follows. The related work is reviewed in Section 2. The network model and the related assumptions are presented in Section 3. We formulate the outage probability minimization problem as an MDP in Section 4. The proposed policies and the related theorems are illustrated in Section 5. The performance and characteristics of the proposed policies are evaluated through numerical results in Section 6. Finally, we conclude this paper in Section 7.

## 2. Related Work

In the literature, the topic of energy harvesting and cognitive radio receive increasing attention. Three groups of existing works are most related. First, the CR technique has received significant attention during the past few years [18,19,20,21,22]. In [18], the authors focus on designing a database access strategy that allows the SUs to jointly consider the requirements of the existing rules, as well as the maximization of the expected communication opportunities through on-demand database access. The optimal strategy introduced in [18], which is computationally unfeasible with the brute-force approach, can be solved by the efficient algorithm proposed in [19]. In [19], by proving that the optimal strategy has a threshold structure, an efficient algorithm is introduced by exploiting the threshold property. In [20], the authors investigate the achievable throughput of an unlicensed sensor network operating over the TV white space spectrum. The achievable throughput is analytically derived as a function of the channel ordering. Additionally, the closed-form expression of the maximum expected throughput is illustrated. The work in [21] studies the problem of coexistence interference among multiple secondary networks without the secondary cooperation. Under a reasonable assumption, a computationally-efficient algorithm for finding the optimal strategy is presented. The work in [22] develops robust power control strategies for cognitive radios in the case of sensing delay and model parameter uncertainty. A robust power control framework that optimizes the worst-case system performance is proposed. All of the problems considered in the above works are formulated as Markov decision process problems, and the technical contributions are very important and valuable. However, due to the unique features of the EH CRSNs, such as the dynamic energy replenishment process, which stipulates a new design constraint on energy usage in the time axis, there is a need to revisit resource allocation policies so that the energy expenditure can efficiently adapt to the dynamics of energy arrivals.

Second, the energy harvesting technique has been widely studied in wireless communication systems [23,24,25,26,27,28,29,30]. The works in [23,24,25,26] consider the point-to-point wireless communications. In [23], through optimizing the time sequence of transmit powers, the authors focus on maximizing the throughput by a deadline and minimizing the transmission completion time. For the offline policy, a directional water-filling algorithm is introduced to find the optimal power allocation. For the online policy, dynamic programming is applied to solve the optimal power allocation. In [24], the authors consider the problem of energy allocation over a finite horizon to maximize the throughput. A water-filling energy allocation where the water-level follows a staircase function is introduced. The work in [25] studies the problem of energy allocation for sensing and transmission to maximize the throughput in an energy harvesting wireless sensor network. The problem studied in [25] considers the finite horizon case, which is extended in [26] to an infinite-horizon case. In [26], the authors study the energy allocation for sensing and transmission for an energy harvesting sensor node. An optimal energy allocation algorithm and an optimal transmission energy allocation algorithm are introduced. The works in [27,28] consider the problem of hybrid energy supply. In [27], the authors investigate the minimization of the power consumption stemming from the constant energy source for transmitting a given number of data packets. In [28], for a hybrid energy supply system employing a save-then-transmit protocol, the authors explore the transmission scheduling problem. In [29], the authors study the transmission power allocation strategy to achieve the energy-efficient transmission. The harvest-use technique is adopted, which means that the harvested energy cannot be stored and must be used immediately. In [30], for a solar-powered wireless sensor network, the authors present an optimal transmission policy based on a data-driven approach. However, due to the distinctive operation of cognitive radio, such as spectrum sensing, spectrum management, etc., directly applying the strategies mentioned above to EH CRSNs can be ineffective or inefficient.

Third, much recent research has been tightly focused on CR systems powered by energy harvesting. The work in [11] focuses on an energy harvesting cognitive radio network with the save-then-transmit protocol; the authors mainly investigate the joint optimization of saving factor, sensing duration, sensing threshold and fusion rules to maximize the achievable throughput. In [31], for a single-user multi-channel setting, jointly considering probabilistic arrival energy, channel conditions and the probability of PU’s occupation, the authors propose a channel selection criterion. In [32], jointly considering the battery replenish process and the secondary belief regarding the primary activities, the authors introduce an energy allocation for sensing and transmission to maximize the long-term throughput. Different from [32], a suboptimal energy allocation algorithm that allocates energy in an online approach is introduced in [33]. In [34], in order to maximize the throughput, the authors derive an optimal sensing strategy through optimizing the access probabilities of idle channels and busy channels. In [35], a joint design of the spectrum sensing and detection threshold to maximize the long-term throughput is studied. Furthermore, the upper bound of the achievable throughput is derived as a function of the energy arrival rate, the statistical behavior of the primary network traffic and the detection threshold in [36]. In [10], the authors propose a spectrum and energy-efficient heterogeneous cognitive radio sensor network (HCRSNs), where EH-enabled spectrum sensors cooperatively detect the status of the licensed channels, while the data sensors transmit data to the sink. Compared with these works, the salient feature of this paper is that, according to the current knowledge of the battery state, channel fading, as well as the arrival energy based on EH, we jointly optimize the action of channel sensing and transmission power allocation for an energy harvesting cognitive sensor node, to minimize the long-term outage probability.

## 3. Network Model

### 3.1. Primary Network Model

We consider a primary network where a primary user (PU) owns the usage right of a channel with bandwidth *B*. The PU is assumed to employ synchronous slotted communication with a time slot duration *T*. The primary traffic is modeled as a two-state time-homogeneous random process, in which the channel randomly switches its state between idle and occupied, as assumed in [34]. Let $\theta_{t}$ represent the status of the channel in time slot *t*, with $\theta_{t}=0$ or 1 indicating that the channel is occupied or idle, respectively. The probability that the channel is occupied by the PU is denoted as $p_{o}≜Pr\left(\theta_{t}=0\right)$. Correspondingly, the idle probability of the channel is defined as $p_{i}≜Pr\left(\theta_{t}=1\right)=1-p_{o}$. It is assumed that $p_{o}$ and $p_{i}$ are available for the secondary users based on the long-term spectrum measurements [35].

### 3.2. Secondary Network Model

#### 3.2.1. Energy Model and Opportunistic Spectrum Access

We consider a point-to-point communication link between two secondary sensor nodes, which are also referred to as secondary users (SUs). An EH-enabled SU opportunistically accesses the primary channel to convey data to its receiver. The EH SU is powered exclusively by energy harvested from the ambient environment (e.g., solar, wind, thermal, ambient radio power) and stores the energy in a rechargeable battery with finite energy storage capacity. A correlated time process following a first-order discrete-time Markovian model is adopted for modeling the energy arrivals [26,37]. According to the harvest-store-use model, the harvested energy in the current time slot can only be used in the next time slot.

**Figure 1:**
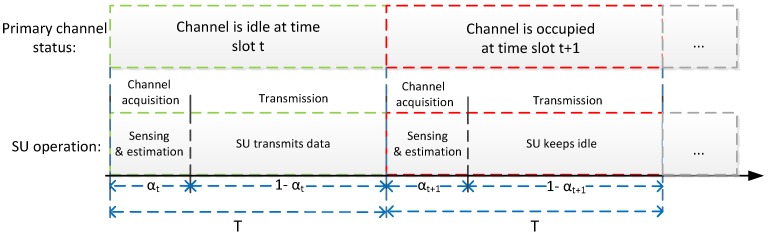
The allocation of time durations: channel acquisition phase versus transmission phase.

Since the PU has priority in utilizing the channel, in order to opportunistically use the channel, the SU has to perform real-time monitoring of the channel to avoid collisions with the PU. Thus, for each time slot, the overall transmission process consists of two phases, namely the channel acquisition phase and the transmission phase. The allocation of time durations for the two phases is illustrated in Figure 1, where the channel acquisition phase and the transmission phase consume $\alpha_{t}$ and $1-\alpha_{t}$ fractions of one time slot, respectively, and $\alpha_{t}$ is referred to as spectrum sensing overhead for time slot *t*, which can be altered to optimize the performance of the system. For the channel acquisition phase, the SU senses the status of the spectrum with $\alpha_{t}T$ time through the energy detection technique [38]. As the complexity is roughly linear in sensing duration, we can assume that the energy consumption $e_{s}$ for sensing is proportional to $\alpha_{t}T$ with a constant sensing power $p_{s}$ [32], namely:

(1) $$e_{s}\left(\alpha_{t}\right)=\alpha_{t}Tp_{s}.$$

If the channel is sensed to be idle, the SU starts data transmission using energy stemming from the battery. Let $P_{t}$ be the transmit power of SU, then the energy consumption for the transmission phase can be expressed as:

(2) $$e_{d}^{1}\left(\alpha_{t},P_{t}\right)=\left(1-\alpha_{t}\right)TP_{t}.$$

If the channel is sensed to be occupied, the SU stays in the idle state with a constant idle power $p_{c}$, which is considerably less than the transmit power [39]; therefore, the energy consumption for the data transmission phase is:

(3) $$e_{d}^{0}\left(\alpha_{t}\right)=\left(1-\alpha_{t}\right)Tp_{c}.$$

#### 3.2.2. Spectrum Sensing and Transmission Data Rate

During the channel acquisition phase mentioned above, the SU acquires the status of the channel by performing a binary hypothesis test to determine between idle $H_{0}$ (i.e., $\theta_{t}=1$) and occupied $H_{1}$ (i.e., $\theta_{t}=0$). Due to the existence of sensing errors, the reliability of spectrum sensing is evaluated by two indicators, namely the false alarm probability $P_{f}$ and the detection probability $P_{d}$, which are defined as follows:

(4) $$P_{f}=Pr\left\{{\hat{\theta }}_{t}=0|\theta_{t}=1\right\},$$

(5) $$P_{d}=Pr\left\{{\hat{\theta }}_{t}=0|\theta_{t}=0\right\},$$

where ${\hat{\theta }}_{t}$ is the binary decision on the primary channel, with ${\hat{\theta }}_{t}=0$ or 1 representing that the primary channel is determined to be occupied or idle, respectively. Considering ensuring sufficient protection to the PU, the SU should satisfy a target detection probability ${\bar{P}}_{d}$ on the primary channel. Regarding the complex-valued primary signal and circularly symmetric complex Gaussian (CSCG) noise case, the probability of a false alarm is given by [40]:

(6) $$P_{f}\left(\alpha_{t}\right)=Q\left(\sqrt{2\beta +1}Q^{-1}\left({\bar{P}}_{d}\right)+\sqrt{\alpha_{t}Tf_{s}}\beta \right),$$

where *β* is the received signal-to-noise ratio (SNR) of the primary signal at SU and $f_{s}$ is the sampling frequency. The function Q(·) is $Q\left(x\right)=\left(1/\sqrt{2\pi }\right)\int_{x}^{\infty }\exp\left(-t^{2}/2\right)dt$.

For time slot *t*, after acquiring the status of the channel, the SU performs channel estimation to obtain the channel condition. Specifically, the SU will send pilot signals to the receiver and acquires the channel power gain, denoted as $\gamma_{t}$, through an error-free and dedicated feedback channel [31]. Since the above channel estimation takes a very short time and limited power, we assume the time and energy consumed in the channel estimation are negligible compared to the sensing time, and hence, we ignore it in our analysis (For example, if PUs are TV bands where each channel occupies 6 MHz in the case of the IEEE802.22 wireless regional area network (WRAN), the typical sensing time is about a few milliseconds, which will result in thousands of samples [40]. However, for channel estimation, a few pilot symbols would be enough. For example, in IEEE802.11a, only four pilot symbols are used for channel estimation [41]), similar to [42,43]. Then, the transmission data rate of the SU is:

(7) $$r\left(\alpha_{t},P_{t},\gamma_{t}\right)=\left(1-\alpha_{t}\right)\log\left(1+\frac{P_{t}\gamma_{t}}{N_{0}}\right),$$

where $N_{0}$ is the destination noise power. The coefficient $1-\alpha_{t}$ is due to the fact that only a $1-\alpha_{t}$ fraction of a time slot is utilized for the SU’s data transmission phase. If, on the other hand, the channel is sensed to be occupied, the sensor node abstains from transmission and stays idle for the rest of the time slot; thus, the transmission data rate $r(\alpha_{t},P_{t},\gamma_{t})$ is zero.

The overall objective of this paper is to design optimal policies by jointly considering the sensing overhead and the transmit power allocation, to minimize the long-term outage probability of the EH-enabled cognitive sensor nodes. In the following section, we will exhibit the procedure of formulating the problem of outage probability minimization as an Markov decision process in detail.

## 4. Problem Formulation

In this section, we formulate the problem of long-term outage probability minimization as an MDP. The MDP model is mainly composed of decision epochs, states, actions, state transition probabilities and rewards. The decision epoch is time slot t∈T={0,1,2,⋯}. The state of the system is denoted as s=(b,g,h), where *b* indicates the battery energy state, *g* indicates the channel state and *h* indicates the state of arrival energy based on EH. We assume that *b*, *g* and *h* take discrete values from discrete finite set $B=\{0,1,2,\cdots ,N_{B}-1\}$, $G=\{0,1,2,\cdots N_{G}-1\}$ and $H=\{0,1,2,\cdots N_{H}-1\}$, respectively. Thus, the state space can be expressed as S=B×G×H, where × denotes the Cartesian product. We assume the battery is quantized in units of $e_{u}$, which can be referred to as one unit of energy quantum . Additionally, we denote the battery energy State 0 corresponds to the energy $B_{0}≜\left\lceil \frac{p_{c}T}{e_{u}}\right\rceil e_{u}$, which is the energy consumption when the SU stays in the idle state within the entire time slot, and for battery state b∈B\{0}, the total energy in the battery is $B_{0}+be_{u}$. As for the arrival energy, if the arrival energy state is h∈H, then the actually arrival energy is $Q_{h}e_{u}$, where $Q_{h}\in N$. It should be noted that as the channel state and arrival energy state can only be acquired casually, at the beginning of time slot *t*, the SU only attains the exact channel state and the arrival energy state of the previous time slot. Therefore, the system state for time slot *t* can be represented as $s_{t}=\left(b_{t},g_{t-1},h_{t-1}\right)$, where $b_{t}\in B$ is the energy state for the current time slot, whereas $g_{t-1}\in G$ and $h_{t-1}\in H$ are the states of channel and arrival energy of the previous time slot. The evolvement of the arrival energy $h_{t}$ is assumed to be a first-order discrete-time Markovian model introduced in Section 3.2; hence, in the following, we will first introduce the update process of the battery energy state $b_{t}$ along with the SU’s channel capacity. Then, the evolvement of the channel state $g_{t}$ is presented.

First, as to the battery energy state update process, a combination of sensing overhead $\alpha_{t}$ and transmit power $P_{t}$ leads to one of the following four possible consequences:

1. Idle detection with probability $p_{i}\left(1-P_{f}\left(\alpha_{t}\right)\right)$: the primary channel is idle while the sensing result is correct. Then, channel capacity:
  (8) $$R=r(\alpha_{t},P_{t},\gamma_{t})$$
  is gained, and the battery energy state updates as:
  (9) $$b_{t+1}=\min\left\{\left\lfloor b_{t}-e_{s}\left(\alpha_{t}\right)/e_{u}-e_{d}^{1}\left(\alpha_{t},P_{t}\right)/e_{u}+Q_{h_{t}}\right\rfloor ,N_{B}-1\right\}.$$
2. False alarm with probability $p_{i}P_{f}\left(\alpha_{t}\right)$: the primary channel is idle while the sensing result is wrong. The SU abstains from the transmission, and the channel capacity *R* is zero. The battery energy state is:
  (10) $$\begin{array}{c} b_{t+1}=\min\left\{\left\lfloor b_{t}-e_{s}\left(\alpha_{t}\right)/e_{u}-e_{d}^{0}\left(\alpha_{t}\right)/e_{u}+Q_{h_{t}}\right\rfloor ,N_{B}-1\right\}. \end{array}$$
3. Occupied detection with probability $p_{o}{\bar{P}}_{d}$: the primary channel is occupied while the sensing result is correct. SU abstains from the transmission, and channel capacity *R* is zero; the battery energy state is the same as (10).
4. Misdetection with probability $p_{o}\left(1-{\bar{P}}_{d}\right)$: the primary channel is occupied while the sensing result is wrong. Channel capacity *R* is zero due to the collision with PU and the battery energy state updates the same as (9).

Second, we formulate the evolvement of channel states. The channel fading process can be modeled as a time-homogeneous finite-state Markov chain (FSMC), which has been widely used to model the block fading channel [44,45,46,47]. Specifically, the channel power is quantized using a finite number of thresholds $G=\{G_{0}=0,G_{1},G_{2},\cdots ,G_{N_{G}}=\infty \}$, where $G_{i}<G_{j}$ when $0\leq i<j\leq N_{G}-1$. The channel is considered to be in state *i*, $0\leq i\leq N_{G}-1$, if the instantaneous channel power gain belongs to the interval $\left[G_{i},G_{i+1}\right)$. We consider that the wireless channel fluctuates slowly over time slots and remains constant within a time slot, as assumed in [48,49]. Hence, the channel state transition occurs only from the current state to its neighboring states at the beginning of each time slot [30]. Considering the Rayleigh fading channel, the channel state transition probability is determined by [50]:

(11) $$P\left(g_{t+1}=j|g_{t}=i\right)=\left\{\begin{array}{ccc} \frac{h(G_{i+1})}{P(g=i)}, & j=i+1,\; & i=0,\ldots ,N_{G}-2;\\ \frac{h(G_{i})}{P(g=i)}, & j=i-1,\; & i=1,\ldots ,N_{G}-1;\\ 1-\frac{h(G_{i})}{P(g=i)}-\frac{h(G_{i+1})}{P(g=i)}, & j=i,\; & i=1,\ldots ,N_{G}-2, \end{array}\right.$$

where P(g=i) is the stationary probability that the channel state is *i*, and $P\left(g=i\right)=\exp\left(-\frac{G_{i}}{G_{a}}\right)-\exp\left(-\frac{G_{i+1}}{G_{a}}\right)$; $G_{a}$ is the average channel power gain. $h\left(\beta \right)=\sqrt{2\pi \beta /G_{a}}f_{D}\exp\left(-\beta /G_{a}\right)$ is the level crossing rate, where $f_{D}$ is the maximum Doppler frequency, normalized by 1/T. The boundary transition probabilities for channel states are:

(12) $$P\left(g_{t+1}=0|g_{t}=0\right)=1-P\left(g_{t+1}=1|g_{t}=0\right),$$

(13) $$P\left(g_{t+1}\;\;=\;\;N_{G}\;-\;1|g_{t}\;\;=\;\;N_{G}\;-\;1\right)\;\;=\;\;1\;\;-\;\;P\left(g_{t+1}\;\;=\;\;N_{G}\;-\;2|g_{t}\;\;=\;\;N_{G}\;-\;1\right).$$

According to the current system state $s_{t}=\left(b_{t},g_{t-1},h_{t-1}\right)$, we introduce the action set of the SU. The sensing overhead $\alpha_{t}$ is quantized in units of $\alpha_{u}=\frac{e_{u}}{p_{s}T}$, and the the action set of sensing overhead can be expressed as follows:

(14) $$A_{\alpha }^{s_{t}}=\left\{\begin{array}{c} \left\{0\right\}\;\;\mathrm{if}\;\;b_{t}=0,\\ \{1,2,\cdots ,\min\left\{\left\lfloor \frac{p_{s}T}{e_{u}}\right\rfloor ,b_{t}\right\}\}\;\;\mathrm{otherwise}, \end{array}\right.$$

where $\left\lfloor \cdot \right\rfloor$ is the floor function. $b_{t}=0$ indicates that the energy level in the battery is so low (the energy stored in the battery is $B_{0}$) that the available energy is merely enough to compensate the energy expenditure when the SU stays in the idle state within the entire time slot. In this case, the SU stops the sensing, as well as transmission and keeps on harvesting energy. Respecting the constraint $\min\{\left\lfloor \frac{P_{s}T}{e_{u}}\right\rfloor ,b_{t}\}$, the first constraint indicates that the sensing duration should be less than the time slot *T*; the second constraint indicates that the energy consumption for sensing should be less than the available energy $b_{t}e_{u}$. When an action $a_{\alpha }\in A_{\alpha }^{s_{t}}$ is taken, the sensing overhead is $a_{\alpha }\cdot \alpha_{u}$, the sensing time is $a_{\alpha }\cdot \alpha_{u}\cdot T=a_{\alpha }\cdot \frac{e_{u}}{P_{s}}$ and the energy consumption for sensing is $e_{s}\left(a_{\alpha }\cdot \alpha_{u}\right)=a_{\alpha }\cdot \alpha_{u}\cdot T\cdot P_{s}=a_{\alpha }e_{u}$. According to the action of sensing overhead, the action set of transmission power is quantized in units of $P_{u}=\frac{e_{u}}{\left(T-a_{\alpha }\alpha_{u}T\right)}$, and the action set can be expressed as:

(15) $$A_{p}^{\left(s_{t},a_{\alpha }\right)}=\left\{0,1,2,\cdots ,b_{t}-a_{\alpha }\right\}.$$

For an action $a_{p}\in A_{p}^{\left(s_{t},a_{\alpha }\right)}$, SU will consume $a_{p}e_{u}$ energy for data transmission.

Therefore, given a system state $s_{t}=\left(b_{t},g_{t-1},h_{t-1}\right)$, the action set can be represented as:

(16) $$A_{s_{t}}=\left\{\left(a_{\alpha },a_{p}\right)|a_{\alpha }\in A_{\alpha }^{s_{t}},a_{p}\in A_{p}^{\left(s_{t},a_{\alpha }\right)}\right\}.$$

We use $P(s_{t+1}|s_{t},a)$ to denote the system state transition probability, which indicates the probability that the system will go into state $s_{t+1}=\left(b_{t+1}=b^{\prime },g_{t}=g^{\prime },h_{t}=h^{\prime }\right)$ in the case that the current system state is $s_{t}=\left(b_{t}=b,g_{t}=g,h_{t}=h\right)$ and SU takes an action $a=\left(a_{\alpha },a_{p}\right)\in A_{s_{t}}$. The state transition probability can be derived as follows:

(17) $$\begin{array}{cc} P(s_{t+1}|s_{t},a) & =P(b^{\prime },g^{\prime },h^{\prime }|b,g,h,a_{\alpha },a_{P})\\ & =P\left(b^{\prime }|b,g^{\prime },h^{\prime },a_{\alpha },a_{P}\right)P\left(g^{\prime }|g\right)P\left(h^{\prime }|h\right) \end{array}$$

where:

(18) $$P(b^{\prime }|b,g^{\prime },h^{\prime },a_{\alpha },a_{P})=\left\{\begin{array}{c} 1\;\;\mathrm{if}\;\;b^{\prime }=\min\left\{\left\lfloor b-a_{\alpha }-I_{a_{P}>0}a_{P}-I_{a_{P}=0}\frac{e_{d}^{0}\left(a_{\alpha }\alpha_{u}\right)}{e_{u}}+Q_{h^{\prime }}\right\rfloor ,N_{B}-1\right\},\\ 0\;\;\mathrm{otherwise}, \end{array}\right.$$

since $b^{\prime }$ is a certain value, which is determined by *b*, $h^{\prime }$ and action $a_{\alpha }$, $a_{p}$. $I_{x}$ denotes the indicator function which takes the value of one if *x* is true, otherwise zero.

The reward function is defined as the outage probability regarding the system state $s_{t}=\left(b_{t},g_{t-1},h_{t-1}\right)$ and the corresponding action $a=(a_{\alpha },a_{p})$, which is given by [51]:

(19) $$\begin{array}{cc} R(s_{t},a) & ≜P_{out}\left(R<R_{th}\right)\\ & =p_{i}\left(1-P_{f}\left(a_{\alpha }\alpha_{u}\right)\right)Pr\left(r\left(a_{\alpha }\alpha_{u},a_{p}P_{u},\gamma_{t}\right)<R_{th}\right)+p_{i}P_{f}\left(a_{\alpha }\alpha_{u}\right)+p_{o}{\bar{P}}_{d}+p_{o}\left(1-{\bar{P}}_{d}\right)\\ & =p_{i}\left(1-P_{f}\left(a_{\alpha }\alpha_{u}\right)\right)\sum_{g_{t}\in G}P\left(g_{t}|g_{t-1}\right)Pr\left(\gamma_{t}<\gamma_{th}|G_{g_{t}}\leq \gamma_{t}<G_{g_{t}+1}\right)\\ & +p_{i}P_{f}\left(a_{\alpha }\alpha_{u}\right)+\left(1-p_{i}\right), \end{array}$$

where $\gamma_{th}=\frac{N_{0}}{a_{p}P_{u}}\left(2^{\frac{R_{th}}{1-a_{\alpha }\alpha_{u}}-1}\right)$. If $\gamma_{th}\geq G_{g_{t}+1}$, then $Pr(\gamma_{t}<\gamma_{th}|G_{g_{t}}\leq \gamma_{t}<G_{g_{t}+1})=1$; if $\gamma_{th}<G_{g_{t}}$, then $Pr(\gamma_{t}<\gamma_{th}|G_{g_{t}}\leq \gamma_{t}<G_{g_{t}+1})=0$; otherwise, $Pr\left(\gamma_{t}<\gamma_{th}|G_{g_{t}}\leq \gamma_{t}<G_{g_{t}+1}\right)=\frac{Pr\{G_{g_{t}}\leq \gamma_{t}<\gamma_{th}\}}{Pr\{G_{g_{t}}\leq \gamma_{t}<G_{g_{t}+1}\}}=\frac{\exp\left(-G_{g_{t}}/G_{a}\right)-\exp\left(-\gamma_{th}/G_{a}\right)}{\exp\left(-G_{g_{t}}/G_{a}\right)-\exp\left(-G_{g_{t}+1}/G_{a}\right)}$.

In the following section, we first mainly study the existence of the optimal transmission policy. Then, the *ϵ*-optimal sensing-transmission policy that specifies the actions concerning the sensing overhead and the transmit power to minimize the long-term outage probability is introduced. Last, for a special case where the signal-to-noise power ratio is sufficiently high, we introduce an efficient transmission policy, which achieves the same performance as the *ϵ*-optimal sensing-transmission policy.

## 5. Proposed Transmission Policies

In this section, we focus on deriving policies that specify the actions regarding the sensing overhead and transmit power, with the goal of minimizing the long-term outage probability. First, we introduce the concept of the stationary deterministic policy. Second, we prove the convergence and the existence of the stationary deterministic policy. Then, based on the Bellman equation, we propose an *ϵ*-optimal stationary deterministic policy named the sensing-transmission policy through the value iteration approach. Last, for the special case where the signal-to-noise (SNR) is sufficiently high, we introduce an efficient transmission policy.

Denote $\pi \left(s\right)=\{d_{0}\left(s_{0}\right),d_{1}\left(s_{1}\right),d_{2}\left(s_{2}\right),\cdots \}$ as the decision policy that specifies the decision rules to be used at each time slot, and $d_{t}$ is the decision rule that prescribes a procedure for action selection in time slot *t*. A policy is stationary deterministic if $d_{t}$ is deterministic Markovian and $d_{t}=d$ for all t∈T [26]; therefore, the stationary deterministic policy can be represented as $\pi \left(s\right)=\{d\left(s_{0}\right),d\left(s_{1}\right),d\left(s_{2}\right),\cdots \}$. For an infinite-horizon MDP, our primary focus will be on the stationary deterministic policy because the decision rules do not change over time, and they are easiest to implement and evaluate [52]. We denote the feasible set of stationary deterministic policies as $\Pi^{SD}$. Given the initial state $s_{0}=\left(b_{0},g_{-1},h_{-1}\right)$ and the policy $\pi \in \Pi^{SD}$, the expected discounted infinite-horizon reward that represents the long-term outage probability is defined to be [52]:

(20) $$V_{\pi }\left(s_{0}\right)=E\left\{\sum_{t=0}^{\infty }\lambda^{t}R\left(s_{t},a\right)|s_{0},\pi \right\},s_{t}\in S,a\in A_{s_{t}},$$

where $V_{\pi }\left(s_{0}\right)$ is the long-term expected reward with respect to the initial state $s_{0}$, 0≤λ<1 is the discount factor, $R(s_{t},a)$ is the reward function defined by (19) and *a* is the action determined by the policy *π*. The alteration of *λ* brings a wide range of performance characteristics, which can be altered according to the actual needs.

The objective of the SU is to find the optimal stationary deterministic policy $\pi^{*}$ that minimize the long-term expected reward defined in (20), that is:

(21) $$\pi^{*}=\min_{\pi \in \Pi^{SD}}V_{\pi }\left(s_{0}\right).$$

First, we prove that the long-term expected reward $V_{\pi }\left(s_{0}\right)$, where $\pi \in \Pi^{SD}$, is finite.

**Lemma** **1.** $V_{\pi }\left(s_{0}\right)$ *is finite, namely* $|V_{\pi }\left(s_{0}\right)|<\infty$*, where* $\pi \in \Pi^{SD}$ *and* $s_{0}\in S$*.*

**Proof of Lemma** **1.** In order to prove that the value of $|V_{\pi }\left(s_{0}\right)|$ is limited, according to [52], we only need to prove that $\underset{a\in A_{s},s\in S}{\sup}\left|R\left(s,a\right)\right|<\infty$. As $Pr(\gamma_{t}<\gamma_{th}|G_{g_{t}}\leq \gamma_{t}<G_{g_{t}+1})\leq 1$, $P(g_{t}|g_{t-1})\leq 1$ and G is discrete and finite, we can deduce that $\sum_{g_{t}\in G}P\left(g_{t}|g_{t-1}\right)Pr\left(\gamma_{t}<\gamma_{th}|G_{g_{t}}\leq \gamma_{t}<G_{g_{t}+1}\right)$ is finite. Since $p_{i}\leq 1$, $P_{f}\leq 1$, it can be derived that |R(s,a)| is limited. Thus, we can conclude that $\underset{a\in A_{s},s\in S}{\sup}\left|R\left(s,a\right)\right|<\infty$, and therefore, $V_{\pi }\left(s_{0}\right)$ is finite. ☐

Lemma 1 indicates that for any initial system state, the value of $V_{\pi }\left(s_{0}\right)$ converges to a certain value. Next, we explain the existence of the optimal stationary deterministic policy $\pi^{*}$.

**Theorem** **1.** *There exists an optimal stationary deterministic policy* $\pi^{*}$ *to minimize the long-term expected reward displayed in Equation (20).*

**Proof of Theorem** **1.** Since the system state S=B×G×H is discrete and finite and for an arbitrary s∈S, the corresponding action space $A_{s}$ is also discrete and finite, thus there exists an optimal stationary deterministic policy [52]. ☐

Given an arbitrary system system *s*, the optimal long-term expected reward $V_{\pi^{*}}\left(s\right)$ should satisfy the following Bellman optimality equation:

(22) $$V_{\pi^{*}}\left(s\right)=\min_{a\in A_{s}}\left\{R\left(s,a\right)+\lambda \sum_{s^{\prime }\in S}P\left(s^{\prime }|s,a\right)V_{\pi^{*}}\left(s^{\prime }\right)\right\},s\in S.$$

The first term on the right-hand side of Equation (22) is the immediate reward for the current time slot, and the second term is the expected total discount future reward if SU chooses action *a*. The well-known value iteration approach is then applied to find the *ϵ*-optimal stationary deterministic policy, as shown in Algorithm 1.
**Algorithm 1** Sensing-transmission (ST) policy.1:Set $V_{0}\left(s\right)=0$ for all s∈S, set i=0, specify ϵ>0.2:For each s∈S, calculate the $V_{i+1}\left(s\right)$ according to$V_{i+1}^{a}\left(s\right)=\left\{R\left(s,a\right)+\lambda \sum_{s^{\prime }\in S}P\left(s^{\prime }|s,a\right)V_{i}\left(s^{\prime }\right)\right\}$, $a\in A_{s}$,$V_{i+1}\left(s\right)=\min_{a\in A_{s}}\left\{V_{i+1}^{a}\left(s\right)\right\}$.3:If $∥V_{i+1}-V_{i}∥<\epsilon \left(1-\lambda \right)/2\lambda$, go to Step 4. Otherwise, increase *i* by 1 and go back to Step 2.4:For each s∈S, choose $d\left(s\right)=\underset{a\in A_{s}}{\mathrm{arg min}}\left\{R\left(s,a\right)+\lambda \sum_{s^{\prime }\in S}P\left(s^{\prime }|s,a\right)V_{i+1}\left(s^{\prime }\right)\right\}$5:Obtain the *ϵ*-optimal transmission policy $\pi_{\epsilon }^{*}=\left\{d,d,\cdots \right\}$

In Algorithm 1, the SU iteratively finds the optimal policy. Specifically, in Step 1, $V_{0}\left(s\right)$ is initialized to zero for all s∈S; the error bound *ϵ* is specified; and set the iteration sequence *i* to be zero. In Step 2, we compute the $V_{i+1}\left(s\right)$ for each s∈S according to the knowledge of $V_{i}\left(s\right)$. Then, in Step 3, the SU first estimates whether $∥V_{i+1}-V_{i}∥<\epsilon \left(1-\lambda \right)/2\lambda$ holds, where $V_{i+1}=\left\{V_{i+1}\left(s\right),\forall s\in S\right\}$, $V_{i}=\left\{V_{i}\left(s\right),\forall s\in S\right\}$ and $∥V_{i+1}-V_{i}∥=\max_{s\in S}\left|V_{i+1}\left(s\right)-V_{i}\left(s\right)\right|$. If the inequality holds, which means that the value iteration algorithm has converged, then we proceed to Step 4 to obtain the decision rule and then formulate the sensing-transmission policy. Otherwise, we need to go back to Step 2 and continue to perform the iteration. According to Algorithm 1, the SU can pre-compute the policy and records it in a look-up table. Then, based on the specific system state, the SU can check the look-up table to find out the corresponding action.

As to the convergence, $V_{i}\left(s\right)$ computed by Step 2 converges to $V_{\pi^{*}}\left(s\right)$ for all s∈S. Once the inequality condition in Step 3 is satisfied, then the obtained optimal policy ensures that $∥V_{\pi_{\epsilon }^{*}}-V_{\pi^{*}}∥<\epsilon$, where $V_{\pi_{\epsilon }^{*}}=\left\{V_{\pi_{\epsilon }^{*}}\left(s\right),\forall s\in S\right\}$ is the long-term expected reward achieved by the *ϵ*-optimal policy obtained in Step 5 of the Algorithm 1. In practice, according to the actual needs, SU can predefine the value of *ϵ* to control the accuracy of convergence. Choosing *ϵ* small enough ensures that the algorithm stops with a policy that is very close to optimal. Next, we introduce the complexity of Algorithm 1. The complexity of each iteration in the value iteration algorithm is $O(N_{state}N_{state}^{\prime }N_{action})$ [53], where $N_{state}$ represents the total number of states in the state space, $N_{state}^{\prime }$ indicates the total number of states that the system can possibly transmit to and $N_{action}$ represents the total number of actions in the action space. For our MDP problem, the total number of states in state space S is $N_{B}\cdot N_{G}\cdot N_{H}$. As the battery state of the next time slot is deterministic and the channel can only transmit to the neighbor state or remains in its current state, therefore the total possible states the current system state can transmit to is $3N_{H}$. The maximum number of actions regarding the sensing overhead, as well as the transmit power is $\left(N_{B}+1\right)N_{B}/2$. Hence, the complexity of each iteration in Algorithm 1 is $O(N_{B}^{3}N_{H}^{2}N_{G})$.

Next, we study the structural property of the proposed sensing-transmission policy. Regarding the reward function, we have the following lemma:

**Lemma** **2.** *Given a system s, for an arbitrary certain action of* $a_{\alpha }$*, the immediate reward* $R(s,a_{\alpha },a_{p})$ *is non-increasing with* $a_{p}$*, namely* $R\left(s,a_{\alpha },a_{p}+1\right)\leq R\left(s,a_{\alpha },a_{p}\right)$*, where* $a_{\alpha }\in A_{\alpha }^{s},a_{p}$ *and* $a_{p}+1\in A_{p}^{\left(s,a_{\alpha }\right)}$*.*

**Proof of Lemma** **2.** First, we prove that for a certain action of $a_{\alpha }$, $Pr(\gamma_{t}<\gamma_{th}\left(a_{p}\right)|G_{g^{\prime }}\leq \gamma_{t}<G_{g^{\prime }+1})$ defined in Equation (19) is non-increasing with transmit action $a_{p}$, namely $Pr\left(\gamma_{t}<\gamma_{th}\left(a_{p}+1\right)|G_{g^{\prime }}\leq \gamma_{t}<G_{g^{\prime }+1}\right)\leq Pr\left(\gamma_{t}<\gamma_{th}\left(a_{p}\right)|G_{g^{\prime }}\leq \gamma_{t}<G_{g^{\prime }+1}\right)$, where $g^{\prime }\in G$. As $\gamma_{th}\left(a_{p}\right)$ is decreasing with $a_{p}$, we have $\gamma_{th}\left(a_{p}+1\right)<\gamma_{th}\left(a_{p}\right)$. If $\gamma_{th}\left(a_{p}\right)>\gamma_{th}\left(a_{p}+1\right)\geq G_{g^{\prime }+1}$, we can derive that $Pr\left(\gamma_{t}<\gamma_{th}\left(a_{p}+1\right)|G_{g^{\prime }}\leq \gamma_{t}<G_{g^{\prime }+1}\right)=Pr\left(\gamma_{t}<\gamma_{th}\left(a_{p}\right)|G_{g^{\prime }}\leq \gamma_{t}<G_{g^{\prime }+1}\right)=1$. If $G_{g^{\prime }}>\gamma_{th}\left(a_{p}\right)>\gamma_{th}\left(a_{p}+1\right)$, we can derive that $Pr\left(\gamma_{t}<\gamma_{th}\left(a_{p}+1\right)|G_{g^{\prime }}\leq \gamma_{t}<G_{g^{\prime }+1}\right)\leq Pr\left(\gamma_{t}<\gamma_{th}\left(a_{p}\right)|G_{g^{\prime }}\leq \gamma_{t}<G_{g^{\prime }+1}\right)=0$. Otherwise, it can be derived that $Pr\left(\gamma_{t}<\gamma_{th}\left(a_{p}+1\right)|G_{g^{\prime }}\leq \gamma_{t}<G_{g^{\prime }+1}\right)<Pr\left(\gamma_{t}<\gamma_{th}\left(a_{p}\right)|G_{g^{\prime }}\leq \gamma_{t}<G_{g^{\prime }+1}\right)$. Therefore, we can conclude that $Pr(\gamma_{t}<\gamma_{th}\left(a_{p}\right)|G_{g^{\prime }}\leq \gamma_{t}<G_{g^{\prime }+1})$ is non-increasing with transmit action $a_{p}$.

Next, we calculate the difference between $R(s,a_{\alpha },a_{p})$ and $R(s,a_{\alpha },a_{p}+1)$:

(23) $$\begin{array}{c} R\left(s,a_{\alpha },a_{p}\right)-R\left(s,a_{\alpha },a_{p}+1\right)\\ =p_{i}\left(1-P_{f}\left(a_{\alpha }\alpha_{u}\right)\right)\sum_{g^{\prime }\in G}P\left(g^{\prime }|g\right)[Pr\left(\gamma_{t}<\gamma_{th}\left(a_{p}\right)|G_{g^{\prime }}\leq \gamma_{t}<G_{g^{\prime }+1}\right)\\ -Pr(\gamma_{t}<\gamma_{th}\left(a_{p}+1\right)|G_{g^{\prime }}\leq \gamma_{t}<G_{g^{\prime }+1})], \end{array}$$

since $Pr(\gamma_{t}<\gamma_{th}\left(a_{p}\right)|G_{g^{\prime }}\leq \gamma_{t}<G_{g^{\prime }+1})$ is non-increasing with $a_{p}$, we can derive that $R\left(s,a_{\alpha },a_{p}\right)-R\left(s,a_{\alpha },a_{p}+1\right)\geq 0$, that is $R\left(s,a_{\alpha },a_{p}+1\right)\leq R\left(s,a_{\alpha },a_{p}\right)$. ☐

**Lemma** **3.** *For any given channel state* g∈G *and arrival energy state* h∈H*, the minimum immediate reward* R(s,a) *is non-increasing in battery state* b∈B*. That is,* $\min_{a^{+}\in A_{s^{+}}}\left\{R(s^{+},a^{+})\right\}\leq \min_{a\in A_{s}}\left\{R(s,a)\right\}$*, where* $s^{+}=\left\{b+1,g,h\right\}$*,* s={b,g,h}*,* $\forall b\in B\backslash \{N_{B}-1\}$*,* g∈G,h∈H*.*

**Proof of Lemma** **3.** The action set for $s^{+}$ can be expressed as $A_{s^{+}}=\left\{\left(a_{\alpha }^{+},a_{p}^{+}\right)|a_{\alpha }^{+}\in A_{\alpha }^{s^{+}},a_{p}^{+}\in A_{p}^{\left(s^{+},a_{\alpha }^{+}\right)}\right\}$, and the action set for *s* can be expressed as $A_{s}=\left\{\left(a_{\alpha },a_{p}\right)|a_{\alpha }\in A_{\alpha }^{s},a_{p}\in A_{p}^{\left(s,a_{\alpha }\right)}\right\}$. When $a_{\alpha }^{+}=a_{\alpha }=w$, we can derive that the unit of transmit power $\frac{e_{u}}{T-a_{\alpha }^{+}\alpha_{u}T}=\frac{e_{u}}{T-a_{\alpha }\alpha_{u}T}$, and $A_{p}^{\left(s^{+},a_{\alpha }^{+}\right)}=\left\{0,1,2,\cdots ,\max\left\{b+1-w,0\right\}\right\}⊇A_{p}^{\left(s,a_{\alpha }\right)}=\left\{0,1,2,\cdots ,\max\left\{b-w,0\right\}\right\}$; according to Lemma 2, we have $\min_{a_{p}^{+}\in A_{p}^{\left(s^{+},a_{\alpha }^{+}\right)}}R\left(s^{+},w,a_{p}^{+}\right)=R\left(s^{+},w,b+1-w\right)$, and $\min_{a_{p}\in A_{p}^{\left(s,a_{\alpha }\right)}}R\left(s,w,a_{p}\right)=R\left(s,w,b-w\right)$. Since:

(24) $$\begin{array}{c} R\left(s^{+},w,b+1-w\right)-R\left(s,w,b-w\right)\\ =p_{i}\left(1-P_{f}\left(a_{\alpha }\alpha_{u}\right)\right)\sum_{g^{\prime }\in G}P\left(g^{\prime }|g\right)[Pr\left(\gamma_{t}<\gamma_{th}\left(b+1-w\right)|G_{g^{\prime }}\leq \gamma_{t}<G_{g^{\prime }+1}\right)\\ -Pr(\gamma_{t}<\gamma_{th}\left(b-w\right)|G_{g^{\prime }}\leq \gamma_{t}<G_{g^{\prime }+1})]\\ \leq 0, \end{array}$$

therefore, we have $\min_{a_{p}^{+}\in A_{p}^{\left(s^{+},a_{\alpha }^{+}\right)}}R\left(s^{+},w,a_{p}^{+}\right)\leq \min_{a_{p}\in A_{p}^{\left(s,a_{\alpha }\right)}}R\left(s,w,a_{p}\right)$.

As $\min\left\{\left\lfloor \frac{P_{s}T}{e_{u}}\right\rfloor ,b+1\right\}\geq \min\left\{\left\lfloor \frac{P_{s}T}{e_{u}}\right\rfloor ,b\right\}$, thus $A_{\alpha }^{s^{+}}⊇A_{\alpha }^{s}$; therefore, we have:

(25) $$\min_{a_{\alpha }^{+}\in A_{\alpha }^{s^{+}}}\min_{a_{p}^{+}\in A_{p}^{\left(s^{+},a_{\alpha }^{+}\right)}}R\left(s^{+},a_{\alpha }^{+},a_{p}^{+}\right)\leq \min_{a_{\alpha }\in A_{\alpha }^{s}}\min_{a_{p}\in A_{p}^{\left(s,a_{\alpha }\right)}}R\left(s,a_{\alpha },a_{p}\right),$$

namely $\min_{a^{+}\in A_{s^{+}}}\left\{R(s^{+},a^{+})\right\}\leq \min_{a\in A_{s}}\left\{R(s,a)\right\}$. ☐

Based on Lemma 3, we have following lemma:

**Lemma** **4.** *For any given channel state* g∈G *and arrival energy state* h∈H*, we have that* $V_{i}\left(b,g,h\right)$ *is non-increasing in the battery state* b∈B*, that is* $V_{i}\left(b+1,g,h\right)\leq V_{i}\left(b,g,h\right)$ $\forall b\in B\backslash \{N_{B}-1\}$*.*

**Proof of Lemma** **4.** We prove this lemma by the induction. When i=1, as the initial condition $V_{0}\left(s\right)=0$ for all s∈S, thus $V_{1}\left(s\right)=\min_{a\in A_{s}}\left\{R(s,a)\right\}$. According to Lemma 3, we have $V_{1}\left(b+1,g,h\right)\leq V_{1}\left(b,g,h\right)$. Assume when i=k, for any given g∈G, h∈H and $\forall b\in B\backslash \{N_{B}-1\}$, $V_{k}\left(b+1,g,h\right)\leq V_{k}\left(b,g,h\right)$ holds. When i=k+1, we use $s^{+}$ to indicate system state (b+1,g,h) and use *s* to indicate system state (b,g,h). The action sets for $s^{+}$ and *s* are $A_{s^{+}}=\left\{\left(a_{\alpha }^{+},a_{p}^{+}\right)|a_{\alpha }^{+}\in A_{\alpha }^{s^{+}},a_{p}^{+}\in A_{p}^{\left(s^{+},a_{\alpha }^{+}\right)}\right\}$ and $A_{s}=\left\{\left(a_{\alpha },a_{p}\right)|a_{\alpha }\in A_{\alpha }^{s},a_{p}\in A_{p}^{\left(s,a_{\alpha }\right)}\right\}$, respectively. When $a_{\alpha }^{+}=a_{\alpha }=w$, for arbitrary $a_{p}^{+}=a_{p}=m$, we have R(b+1,g,h,w,m)=R(b,g,h,w,m). Since $\min\left\{\left\lfloor b+1-w-I_{m>0}m-I_{m=0}\frac{e_{d}^{0}\left(w\alpha_{u}\right)}{e_{u}}+Q_{h}\right\rfloor ,N_{B}-1\right\}\geq \min\left\{\left\lfloor b-w-I_{m>0}m-I_{m=0}\frac{e_{d}^{0}\left(w\alpha_{u}\right)}{e_{u}}+Q_{h}\right\rfloor ,N_{B}-1\right\}$, for any g∈G,h∈H, we have that:

(26) $$\begin{array}{c} V_{k}\left(\min\left\{\left\lfloor b+1-w-I_{m>0}m-I_{m=0}\frac{e_{d}^{0}\left(w\alpha_{u}\right)}{e_{u}}+Q_{h}\right\rfloor ,N_{B}-1\right\},g,h\right)\leq \\ V_{k}\left(\min\left\{\left\lfloor b-w-I_{m>0}m-I_{m=0}\frac{e_{d}^{0}\left(w\alpha_{u}\right)}{e_{u}}+Q_{h}\right\rfloor ,N_{B}-1\right\},g,h\right). \end{array}$$

Since $A_{p}^{\left(s^{+},w\right)}⊇A_{p}^{\left(s,w\right)}$, we can deduce that:

(27) $$\begin{array}{c} \min_{a_{p}^{+}\in A_{p}^{s^{+}}}\{R\left(b+1,g,h,w,a_{p}^{+}\right)+\\ \lambda \sum_{h^{\prime }\in H}\sum_{g^{\prime }\in G}P\left(h^{\prime }|h\right)P\left(g^{\prime }|g\right)V_{k}\left(\min\left\{\left\lfloor b+1-w-I_{a_{p}^{+}>0}a_{p}^{+}-I_{a_{p}^{+}=0}\frac{e_{d}^{0}\left(w\alpha_{u}\right)}{e_{u}}+Q_{h^{\prime }}\right\rfloor ,N_{B}-1\right\},g^{\prime },h^{\prime }\right)\}\\ \leq \min_{a_{p}\in A_{p}^{s}}\{R\left(b,g,h,w,a_{p}\right)+\\ \lambda \sum_{h^{\prime }\in H}\sum_{g^{\prime }\in G}P\left(h^{\prime }|h\right)P\left(g^{\prime }|g\right)V_{k}\left(\min\left\{\left\lfloor b-w-I_{a_{p}>0}a_{p}-I_{a_{p}=0}\frac{e_{d}^{0}\left(w\alpha_{u}\right)}{e_{u}}+Q_{h^{\prime }}\right\rfloor ,N_{B}-1\right\},g^{\prime },h^{\prime }\right)\}. \end{array}$$

As $A_{\alpha }^{s^{+}}⊇A_{\alpha }^{s}$, we have:

(28) $$\begin{array}{c} V_{k+1}\left(b+1,g,h\right)=\\ \min_{a_{\alpha }^{+}\in A_{\alpha }^{s^{+}}}\min_{a_{p}^{+}\in A_{p}^{s^{+}}}\{R\left(b+1,g,h,a_{\alpha }^{+},a_{p}^{+}\right)+\\ \lambda \sum_{h^{\prime }\in H}\sum_{g^{\prime }\in G}P\left(h^{\prime }|h\right)P\left(g^{\prime }|g\right)V_{k}\left(\min\left\{b+1-a_{\alpha }^{+}-I_{a_{p}^{+}>0}a_{p}^{+}-I_{a_{p}^{+}=0}\frac{e_{d}^{0}\left(w\alpha_{u}\right)}{e_{u}}+Q_{h^{\prime }},N_{B}-1\right\},g^{\prime },h^{\prime }\right)\}\\ \leq \min_{a_{\alpha }\in A_{\alpha }^{s}}\min_{a_{p}\in A_{p}^{s}}\{R\left(b,g,h,a_{\alpha },a_{p}\right)+\\ \lambda \sum_{h^{\prime }\in H}\sum_{g^{\prime }\in G}P\left(h^{\prime }|h\right)P\left(g^{\prime }|g\right)V_{k}\left(\min\left\{b-a_{\alpha }-I_{a_{p}>0}a_{p}-I_{a_{p}=0}\frac{e_{d}^{0}\left(w\alpha_{u}\right)}{e_{u}}+Q_{h^{\prime }},N_{B}-1\right\},g^{\prime },h^{\prime }\right)\}\\ =V_{k+1}\left(b,g,h\right). \end{array}$$

☐

According to Lemma 4, we have the following theorem:

**Theorem** **2.** *For any given channel state* g∈G *and arrival energy state* h∈H*, the long-term expected reward achieved by the proposed sensing-transmission policy is non-increasing in the battery state b, that is* $V_{\pi_{\epsilon }^{*}}\left(b+1,g,h\right)\leq V_{\pi_{\epsilon }^{*}}\left(b,g,h\right)$, $\forall b\in B\backslash \{N_{B}-1\}$*.*

**Proof of Theorem** **2.** Assume when i=k, the inequality $∥V_{k+1}-V_{k}∥<\epsilon \left(1-\lambda \right)/2\lambda$ holds. According to Step 4 in Algorithm 1, $V_{\pi_{\epsilon }^{*}}\left(s\right)$ is actually $V_{k+2}\left(s\right)$. Based on Lemma 3, we can conclude that $V_{k+2}\left(b+1,g,h\right)\leq V_{k+2}\left(b,g,h\right)$, namely $V_{\pi_{\epsilon }^{*}}\left(b+1,g,h\right)\leq V_{\pi_{\epsilon }^{*}}\left(b,g,h\right)$, $\forall b\in B\backslash \{N_{B}-1\}$. ☐

**Figure 2:**
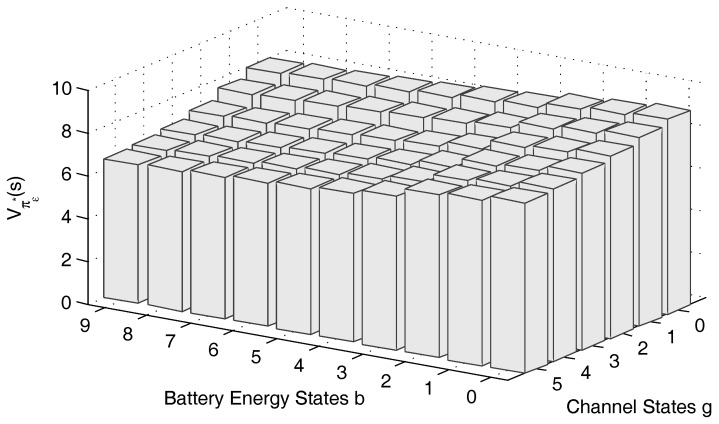
Long-term expected reward $V_{\pi_{\epsilon }^{*}}\left(s\right)$ with battery energy states and channel states. The arrival energy state is h=1, and the number of battery energy states is $N_{B}=10$.

From Theorem 2, we perceive that the long-term reward $V_{\pi_{\epsilon }^{*}}\left(s\right)$ is non-increasing in the battery state *b*. By taking the parameters in Section 6 except as otherwise stated, the reward of the proposed *ϵ*-optimal sensing-access policy is depicted in Figure 2. From Figure 2, we can see that $V_{\pi_{\epsilon }^{*}}\left(s\right)$ is non-increasing in the direction along the battery state, which validates Theorem 2.

**Theorem** **3.** *For any given channel state* g∈G *and arrival energy state* h∈H*, the optimal long-term expected reward achieved by optimal policy* $\pi^{*}$ *is non-increasing in battery state b, that is* $V_{\pi^{*}}\left(b+1,g,h\right)\leq V_{\pi^{*}}\left(b,g,h\right)$*,* $\forall b\in B\backslash \{N_{B}-1\}$*.*

**Proof of Theorem** **3.** According to Theorem 2, we acquire that the *ϵ*-optimal policy is non-increasing in battery state *b*; therefore, the optimal long-term expected reward $V_{\pi^{*}}\left(b,g,h\right)=\lim_{\epsilon \to 0}V_{\pi_{\epsilon }^{*}}\left(b,g,h\right)$ is non-increasing in *b* for any given *g* and *h*. ☐

In the following, we consider a special case where the signal-to-noise ratio (SNR) is sufficiently high. When SNR is sufficiently high, namely $N_{0}\to 0$, the reward function for the system state s=(b,g,h) and the corresponding action $a=(a_{\alpha },a_{p})$ are degenerated to:

(29) $$\lim_{N_{0}\to 0}R\left(s,a\right)=\left\{\begin{array}{cc} 1,\; & a_{p}=0,\\ p_{i}P_{f}\left(a_{\alpha }\alpha_{u}\right)+1-p_{i}, & a_{p}\geq 1,a_{\alpha }\geq 1. \end{array}\right.$$

For the *i*-th iteration, denote the long-term expected reward function with respect to action $a=(a_{\alpha },a_{p})$ as $V_{i}^{\left(a_{\alpha },a_{p}\right)}$. Then, we have the following theorem.

**Theorem** **4.** *When the SNR is sufficiently high, for any iteration i, the expected reward with action* $a=(a_{\alpha },1)$ *is no greater than the expected reward with action* $a=(a_{\alpha },a_{p})$*, where* $a_{p}\geq 1$*. That is,* $V_{i}^{\left(a_{\alpha },1\right)}\left(s\right)\leq V_{i}^{\left(a_{\alpha },a_{p}\right)}\left(s\right)$*, where* $1\leq a_{p}\in A_{p}^{\left(s,a_{\alpha }\right)}$*.*

**Proof of Theorem** **4.** The value difference of the two long-term expected rewards with actions $a=(a_{\alpha },1)$ and $a=(a_{\alpha },a_{p})$ can be calculated as:

(30) $$\begin{array}{c} V_{i+1}^{\left(a_{\alpha },1\right)}\left(s\right)-V_{i+1}^{\left(a_{\alpha },a_{p}\right)}\left(s\right)\\ =p_{i}P_{f}\left(a_{\alpha }\alpha_{u}\right)+1-p_{i}+\lambda \sum_{h^{\prime }\in H}\sum_{g^{\prime }\in G}P\left(h^{\prime }|h\right)P\left(g^{\prime }|g\right)V_{i}\left(b_{a_{\alpha }+1}^{\prime },g^{\prime },h^{\prime }\right)-\\ p_{i}P_{f}\left(a_{\alpha }\alpha_{u}\right)+1-p_{i}+\lambda \sum_{h^{\prime }\in H}\sum_{g^{\prime }\in G}P\left(h^{\prime }|h\right)P\left(g^{\prime }|g\right)V_{i}\left(b_{a_{\alpha }+a_{p}}^{\prime },g^{\prime },h^{\prime }\right)\\ =\lambda \sum_{h^{\prime }\in H}\sum_{g^{\prime }\in G}P\left(h^{\prime }|h\right)P\left(g^{\prime }|g\right)\left[V_{i}\left(b_{a_{\alpha }+1}^{\prime },g^{\prime },h^{\prime }\right)-V_{i}\left(b_{a_{\alpha }+a_{p}}^{\prime },g^{\prime },h^{\prime }\right)\right], \end{array}$$

where $b_{x}^{\prime }=\min\left\{b-x+Q_{h^{\prime }},N_{B}-1\right\}$. As $b_{a_{\alpha }+1}^{\prime }=\min\left\{b-a_{\alpha }-1+Q_{h^{\prime }},N_{B}-1\right\}\geq \min\left\{b-a_{\alpha }-a_{p}+Q_{h^{\prime }},N_{B}-1\right\}=b_{a_{\alpha }+a_{p}}^{\prime }$, according to Lemma 4, we have $V_{i}\left(b_{a_{\alpha }+1}^{\prime },g^{\prime },h^{\prime }\right)-V_{i}\left(b_{a_{\alpha }+a_{p}}^{\prime },g^{\prime },h^{\prime }\right)\leq 0$; thus, we can derive $V_{i+1}^{\left(a_{\alpha },1\right)}\left(s\right)-V_{i+1}^{\left(a_{\alpha },a_{p}\right)}\left(s\right)\leq 0$. ☐

Based on Theorem 4, we can deduce the following theorem:

**Theorem** **5.** *When the SNR is sufficiently high, for any iteration i with a certain action of sensing overhead* $a_{\alpha }$*, the action set of transmit power to minimize the expected reward is* $A_{p_{new}}^{\left(s,a_{\alpha }\right)}=\left\{0,\min\left\{1,b-a_{\alpha }\right\}\right\}$*, where* $a_{\alpha }\in A_{\alpha }^{s}$*,* s∈S*.*

**Proof of Theorem** **5.** When b≤1, the available transmit power set is $\left\{0\right\}\in A_{p_{new}}^{\left(s,a_{\alpha }\right)}=\left\{0\right\}$. When b=2, if $a_{\alpha }=1$, the available transmit power set is $\left\{1\right\}\in A_{p_{new}}^{\left(s,a_{\alpha }\right)}=\left\{0,\min\left\{1,1\right\}\right\}=\left\{0,1\right\}$; otherwise $a_{\alpha }=2$; the available transmit power set is $\left\{0\right\}\in A_{p_{new}}^{\left(s,a_{\alpha }\right)}=\left\{0\right\}$. When b≥3, we have two cases:

- Case 1: $\min\{\left\lfloor \frac{p_{s}T}{e_{u}}\right\rfloor ,b\}=b$, then if $a_{\alpha }=b$, the action set is $\left\{0\right\}\in A_{p_{new}}^{\left(s,a_{\alpha }\right)}=\left\{0\right\}$; otherwise, for arbitrary $a_{\alpha }\in A_{\alpha }^{s}\backslash \left\{b\right\}\leq b-1$, according to Theorem 4, we have $V_{i}^{\left(a_{\alpha },1\right)}\left(s\right)\leq V_{i}^{\left(a_{\alpha },a_{p}\right)}\left(s\right)$ where $a_{p}\geq 1$; therefore, the transmit power set to minimize the long-term value $V_{i}^{\left(a_{\alpha },1\right)}\left(s\right)$ is $\left\{0,1\right\}=A_{p_{new}}^{\left(s,a_{\alpha }\right)}=\left\{0,1\right\}$.
- Case 2: $\min\{\left\lfloor \frac{p_{s}T}{e_{u}}\right\rfloor ,b\}<b$, for arbitrary $a_{\alpha }\in A_{\alpha }^{s}\leq b-1$, according to Theorem 4, we have $V_{i}^{\left(a_{\alpha },1\right)}\left(s\right)\leq V_{i}^{\left(a_{\alpha },a_{p}\right)}\left(s\right)$ where $a_{p}\geq 1$; therefore, the action set to minimize the long-term value $V_{i}^{\left(a_{\alpha },1\right)}\left(s\right)$ is $\left\{0,1\right\}=A_{p_{new}}^{\left(s,a_{\alpha }\right)}=\left\{0,1\right\}$.

Thus, we can derive that the action set to minimize the long-term reward is $A_{p_{new}}^{\left(s,a_{\alpha }\right)}=\left\{0,\min\left\{1,b-a_{\alpha }\right\}\right\}$. ☐

Based on Theorem (5), we present an efficient transmission policy with reduced computational complexity, which is suitable for the case that the SNR is sufficiently high, as shown in Algorithm 2.
**Algorithm 2** Efficient transmission (ET) policy.1:Set $V_{0}\left(s\right)=0$ for all s∈S, set i=0, specify ϵ>0.2:For each s=(b,g,h)∈S, formulate the new action space:$A_{\alpha }^{s}=\left\{\begin{array}{c} \left\{0\right\}\;\;\mathrm{if}\;\;b_{t}=0,\\ \{1,2,\cdots ,\min\left\{\left\lfloor \frac{p_{s}T}{e_{u}}\right\rfloor ,b_{t}\right\}\}\;\;\mathrm{otherwise}, \end{array}\right.$$A_{p_{new}}^{\left(s,a_{\alpha }\right)}=\left\{0,\min\left\{1,b-a_{\alpha }\right\}\right\}$,$A_{s_{new}}=\left\{\left(a_{\alpha },a_{p}\right)|a_{\alpha }\in A_{\alpha }^{s},a_{p}\in A_{p_{new}}^{\left(s,a_{\alpha }\right)}\right\}$,Calculate the $V_{i+1}\left(s\right)$ according to$V_{i+1}^{a}\left(s\right)=\left\{R\left(s,a\right)+\lambda \sum_{s^{\prime }\in S}P\left(s^{\prime }|s,a\right)V_{i}\left(s^{\prime }\right)\right\}$, $a\in A_{s_{new}}$,$V_{i+1}\left(s\right)=\min_{a\in A_{s_{new}}}\left\{V_{i+1}^{a}\left(s\right)\right\}$.3:If $∥V_{i+1}-V_{i}∥<\epsilon \left(1-\lambda \right)/2\lambda$, go to Step 4. Otherwise, increase *i* by 1 and go back to step 2.4:For each s∈S, choose $d\left(s\right)=\underset{a\in A_{s_{new}}}{\mathrm{arg min}}\left\{R\left(s,a\right)+\lambda \sum_{s^{\prime }\in S}P\left(s^{\prime }|s,a\right)V_{i+1}\left(s^{\prime }\right)\right\}$5:Obtain the efficient transmission policy $\pi_{\epsilon }^{*}=\left\{d,d,\cdots \right\}$

In Algorithm 2, since $V_{i}^{\left(a_{\alpha },1\right)}\left(s\right)\leq V_{i}^{\left(a_{\alpha },a_{p}\right)}\left(s\right)$ illustrated in Theorem 4, we can ignore the actions that $a_{p}>1$ and formulate the new action space $A_{s_{new}}$ with a lesser number of candidate actions, which reduces the computational complexity significantly. The total number of states in the state space is $N_{B}\cdot N_{G}\cdot N_{H}$. Similar to the analysis of Algorithm 1, the total possible states the current system state can transmit to is $3N_{H}$. The maximum number of actions regarding the sensing overhead is $N_{B}$, and the maximum number of actions regarding the transmit power is two. Therefore, the complexity of each iteration in Algorithm 2 is $O(N_{B}^{2}N_{H}^{2}N_{G})$.

## 6. Numerical Results and Discussion

In this section, we evaluate the performance and characteristics of the proposed policies by extensive simulations on MatlabR2012a. Unless otherwise stated, the system parameters employed in the simulation are summarized in Table 1, which draws mainly from [26,30,31,42]. The unit of the energy quantum is $e_{u}=0.5$ mJ, and $N_{B}=20$. The quantization levels of the channel power are G={0,0.3,0.6,1.0,2.0,3.0}. The arrival energy takes values from the finite set $\left\{0,4e_{u},6e_{u},8e_{u}\right\}$ mJ per time slot, namely $Q_{0}=0$, $Q_{1}=4$, $Q_{2}=6$, $Q_{3}=8$, and evolves according to the four-state Markov chain with the state transition probability given by:

(31) $$P_{h}=\left[\begin{array}{cccc} P_{0,0} & P_{0,1} & P_{0,2} & P_{0,3}\\ P_{1,0} & P_{1,1} & P_{1,2} & P_{1,3}\\ P_{2,0} & P_{2,1} & P_{2,2} & P_{2,3}\\ P_{3,0} & P_{3,1} & P_{3,2} & P_{3,3} \end{array}\right]=\left[\begin{array}{cccc} 0.5 & 0.5 & 0 & 0\\ 0.25 & 0.5 & 0.25 & 0\\ 0 & 0.25 & 0.5 & 0.25\\ 0 & 0 & 0.5 & 0.5 \end{array}\right].$$

A normalized SNR $\gamma_{c}$ (i.e., $\gamma_{c}=1/N_{0}$) is defined with respect to the transmit power of 1 mW throughout the simulation. We choose *ϵ* to be $10^{-2}$. The initial energy state is $b_{0}=6$; the initial channel state is $g_{-1}=1$; and the initial arrival energy state is $h_{-1}=1$. The total simulation duration is 500 time slots. All of the numerical results are averaged over 500 independent runs.

**Figure 3:**
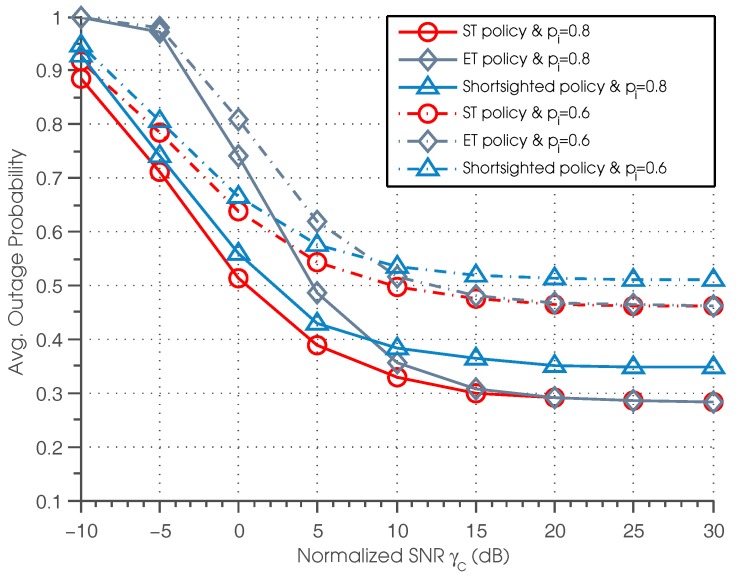
Average outage probability vs. normalized SNR.

**Figure 4:**
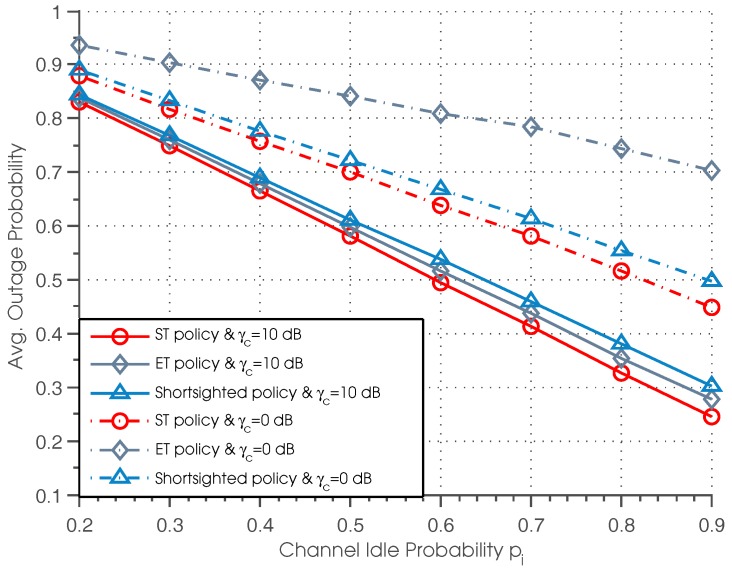
Average outage probability vs. channel idle probability.

**Figure 5:**
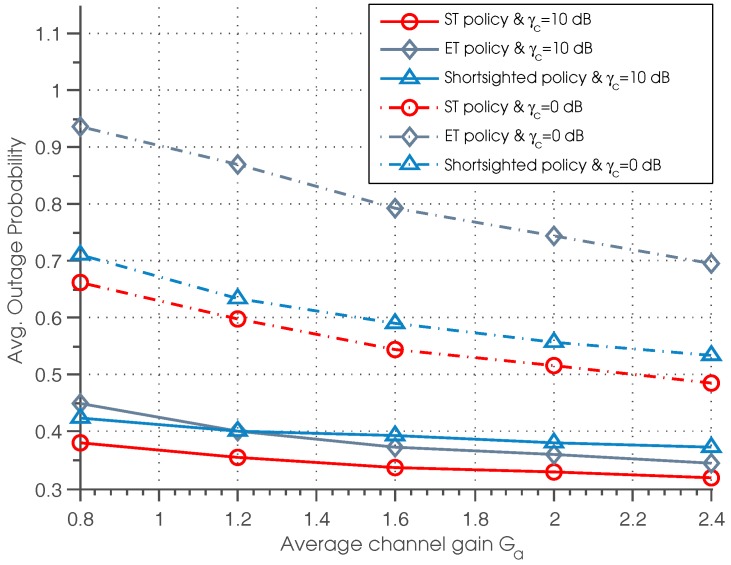
Average outage probability vs. average channel gain.

**Figure 6:**
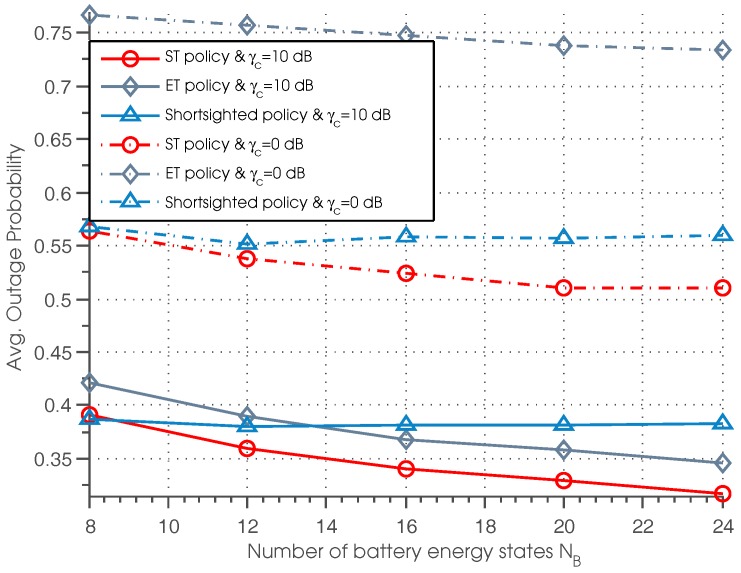
Average outage probability vs. the number of battery energy states.

We compare the proposed sensing-transmission (ST) and efficient transmission (ET) policies with a benchmark named shortsighted policy [32,54] in terms of the performance in Figure 3, Figure 4, Figure 5 and Figure 6. The primary concern of the shortsighted policy is to minimize the immediate reward of the current time slot, without considering the impact of the current action on the future reward, i.e., λ=0. However, the policies proposed in this paper take into account not only the current immediate reward, but also the future expected reward. Therefore, by comparing with the shortsighted policy, we can evaluate the benefit and advantage of proposed policies. Figure 3 depicts the outage probability of ST, ET and the shortsighted policies under different normalized SNRs and channel idle probabilities. First, it can be seen that the ST policy outperforms the shortsighted policy for all settings of normalized SNR. This can be explained by the fact that the ST policy considers a tradeoff between the current immediate reward and the future achievable reward; while the shortsighted policy only focuses on maximizing the current immediate reward, ignoring the impact of the current action on the future reward. It should be noted that despite the better performance of the ST policy, it is much more computationally extensive than the shortsighted policy. Second, we can see that for ST and ET policies, when $\gamma_{c}$ is sufficiently high, the curves of ST and ET policies almost overlap, and a saturation effect is observed, namely the outage probability gradually converges to the same value. This phenomenon coincides with Theorem 5, that is when $\gamma_{c}$ is sufficiently high, the transmit action set that SU needs to consider is $A_{p_{new}}^{s,a_{\alpha }}$, and that ET policy is equivalent to the ST policy in high $\gamma_{c}$ regions. Third, we also observe that the saturation outage probability of the three policies in high SNR regions becomes smaller when $p_{i}$ gets larger. This is because larger $p_{i}$ indicates more probability of employing the licensed channel for data transmission, resulting in lower outage probability.

Figure 4 plots the outage probability of three policies versus the channel idle probability for different values of normalized SNR, where the performance curves plotted correspond to $\gamma_{c}=0$ dB and $\gamma_{c}=10$ dB, respectively. It can be seen that ST policy outperforms the other two policies for all settings of $p_{i}$. Besides, we can observe that the outage probability of all three policies decreases with the increase of channel idle probability, which can be easily understood since a higher value of $p_{i}$ results in a higher possibility of successful data transmission and therefore reduces the outage probability. We can also observe that when $\gamma_{c}$ is small ($\gamma_{c}=0$ dB), the gap between the ST and ET policies becomes larger as $p_{i}$ increases, and the shortsighted policy achieves better performance than the ET policy. While when $\gamma_{c}$ is large ($\gamma_{c}=10$ dB), there is only a tiny difference between the ST and ET policies, and the ET policy achieves better performance than the shortsighted policy.

Figure 5 illustrates the outage probability of three policies as a function of average channel gain $G_{a}$ for different $\gamma_{c}$. It can be observed that the outage probability goes down with the increase of $G_{a}$. This is due to the fact that as $G_{a}$ increases, the data transmission is more efficient when the primary channel is idle, resulting in lower outage probability. Besides, we can see that the ST policy outperforms the other two policies for all of the settings of $G_{a}$. It is also shown that when $\gamma_{c}$ is small ($\gamma_{c}=0$ dB), the shortsighted policy outperforms the ET policy, while when $\gamma_{c}$ is large ($\gamma_{c}=10$ dB), the ET policy achieve better performance than shortsighted performance in the case that $G_{a}\geq 1.2$. Thus, we can conclude that in the case that the $\gamma_{c}$ is small or the channel quality is poor, the shortsighted policy outperforms the ET policy.

Figure 6 plots the outage probability of three policies with different settings of battery energy state and normalized SNR. It can be seen that the outage probability with respect to ST and ET policies decreases as $N_{B}$ increases; while the outage probability regarding the shortsighted policy almost remains unchanged under different values of $N_{B}$. This phenomenon indicates that by increasing the capacity of the battery, we can efficiently decrease the outage probability, but the performance of the shortsighted policy is almost independent of the battery capacity. Besides, we can also observe that for lower $\gamma_{c}$, the performance of shortsighted policy outperforms the ET policy. For higher $\gamma_{c}$, the ET policy achieves better performance than the shortsighted policy when $N_{B}\geq 14$.

**Figure 7:**
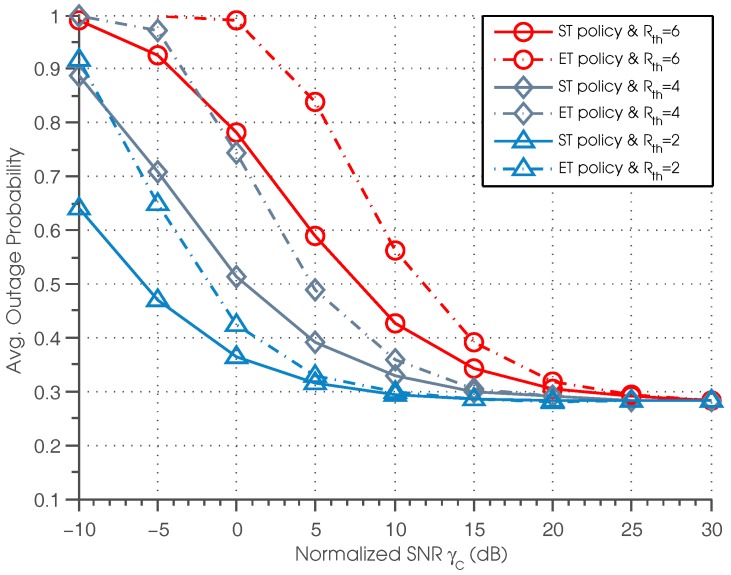
Average outage probability vs. normalized SNR.

Figure 7 shows the outage probability of ST and ET policies as a function of $\gamma_{c}$ for different data rate threshold $R_{th}$. We can see that for lower $\gamma_{c}$, a lower data rate threshold leads to a lower outage probability, and the curves with $R_{th}=2$ outperform the curves with $R_{th}=4$ and $R_{th}=6$. However, when $\gamma_{c}$ is sufficiently high, we observe that the curves correspond to $R_{th}=2$, $R_{th}=4$ and $R_{th}=6$ all converge to the same value. This is because when $\gamma_{c}$ is sufficiently high, according to Equation (29), the reward functions have no relation to $R_{th}$; the curves with different $R_{th}$ achieve the same outage probability.

**Figure 8:**
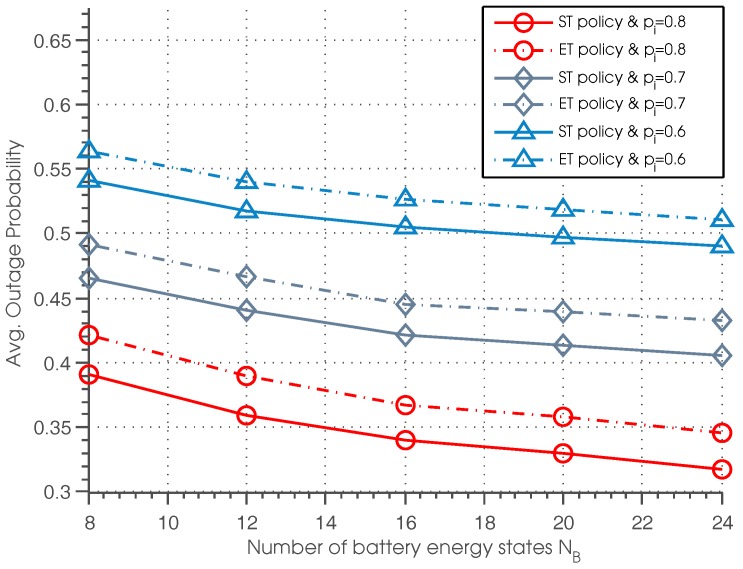
Average outage probability vs. the number of battery energy states.

The outage probability of ST and ET policies with different settings of battery energy state $N_{B}$ and idle probability $p_{i}$ is shown in Figure 8. It can be seen that outage probability of the ST and ET policies decreases as the battery storage capacity $N_{B}$ increases. This is because with a higher $N_{B}$, SU can allocate the energy more efficiently: if the expected channel condition of the next time slot is good and the channel occupancy is estimated to be idle with high probability, the SU can allocate more energy for data transmission; otherwise, the SU can allocate less energy for data transmission and save more energy for future utilization.

## 7. Conclusions

In this paper, we have considered a time-slotted energy harvesting cognitive radio sensor network, where the cognitive sensor nodes solely rely on harvested energy for spectrum sensing and data transmission. Our goal is to minimize the long-term outage probability of the sensor node by adapting the sensing time and transmission power to the current sensor node’s knowledge of battery energy, channel fading and harvested energy. This problem has been formulated as an infinite-horizon discounted MDP . The existence of the optimal stationary deterministic policy has been proven, and an *ϵ*-optimal sensing-transmission policy has been presented through using value iterations. *ϵ* is the error bound between the ST policy and the optimal policy, which can be pre-defined according to the actual need. Moreover, for a special case where the signal-to-noise (SNR) power ratio is sufficiently high, we have introduced an efficient optimal transmission policy with reduced computational complexity. It has been illustrated that the efficient transmission policy is equivalent to the sensing-transmission policy for high regions of SNR. Finally, we have conducted extensive simulations to verify the performance of the proposed policies, and the impacts of system parameters have also been investigated.

## Figures and Tables

**Table 1:** Simulation parameters.

Parameter	Notation	Value
Duration of a time slot	*T*	100 ms
Sampling frequency	$f_{s}$	1 MHz
Channel idle probability	$p_{i}$	0.8
Sensing power	$p_{s}$	100 mw
Target detection probability	$P_{d}$	0.99
Primary signal’s SNR	*β*	−15 dB
Average channel gain	$G_{a}$	2
Normalized Doppler frequency	$f_{D}$	0.05
Discount factor	*λ*	0.99
Normalized SNR	$\gamma_{c}$	10 dB
Data rate threshold	$R_{th}$	4 bits/time slot/Hz
Idle state power	$P_{c}$	3 mw
